# Stigma and Self-Stigma in Addiction

**DOI:** 10.1007/s11673-017-9784-y

**Published:** 2017-05-03

**Authors:** Steve Matthews, Robyn Dwyer, Anke Snoek

**Affiliations:** 1 Plunkett Centre for Ethics, Centre for Moral Philosophy and Applied Ethics, Australian Catholic University (ACU), Institute for Religion and Critical Inquiry (IRCI), 7 Ice Street, Darlinghurst, Sydney, NSW 2010 Australia; 2 Social Studies of Addiction Concepts (SSAC) Research Program, National Drug Research Institute (Melbourne Office), Faculty of Health Sciences, Curtin University, Bentley, Australia; 3 Centre for Cultural Diversity and Wellbeing, College of Arts, Victoria University, Melbourne, Australia; 4 Faculty of Health, Medicine and Life Sciences, Maastricht University, Peter Debyeplein 1, 6229 HA Maastricht, The Netherlands

**Keywords:** Addiction, Self-stigmatization, Shame, Stereotype, Stigma

## Abstract

Addictions are commonly accompanied by a sense of shame or self-stigmatization. Self-stigmatization results from public stigmatization in a process leading to the internalization of the social opprobrium attaching to the negative stereotypes associated with addiction. We offer an account of how this process works in terms of a range of looping effects, and this leads to our main claim that for a significant range of cases public stigma figures in the social construction of addiction. This rests on a social constructivist account in which those affected by public stigmatization internalize its norms. Stigma figures as part-constituent of the dynamic process in which addiction is formed. Our thesis is partly theoretical, partly empirical, as we source our claims about the process of internalization from interviews with people in treatment for substance use problems.

I suppose they think you’re the sort of person going to steal their VCR … ’cause [of] that typical image of a drug addict as some sort of homeless, stinking kind of shambling person who can barely speak and stuff, and I was never like that even when I was using, but that’s the impression. —Adam

## Introduction

Owen Flanagan (2013) has recently proposed an account of addiction that includes a shame condition. “Addicted” persons interpret themselves as both failing in effective agency and not living up to their own normative standards, and their recognition of this leads to a set of negative self-regarding attitudes, central to these being shame. Flanagan thinks, and we agree, that the sources of the shame condition connect to the affected individual’s perceived inability to be an effective reasons-responsive agent, someone, as he says, who passes *her own* survey. However, we think that in addition to this narrow source of shame there is a wider source: the public stigmatization of addiction and of people experiencing addiction.

To be fair to Flanagan, he too includes socially sourced shame in addition to the phenomenon of shame in one’s own eyes. Indeed, he says (2013, 3) that addiction is “ … actually a person-in-a-particular-social-world disorder.” There is, however, the question of emphasis. We claim that a narrow source of shame—a loss of face for failures to live up to one’s own standards—misses much of what explains it, namely the fact that affected persons mark themselves out to themselves—they *self*-stigmatize—after absorbing negative social attitudes about addiction, addictive behaviour and “addicts.” Even the concept of shame in one’s own eyes, where one tries to meet some personal normative standard, is unlikely not to suffer from the leakage of social norms into personal care of oneself. These standards are derived from social learning where we quickly learn that in letting ourselves down we typically let down others who rely on us.

Importantly, we are not claiming that socially induced shame, or self-stigmatization, applies in equally robust proportions to the population of individuals affected by addiction.^1^ Indeed, we are not claiming it applies to all individuals experiencing addiction. Our claim is that for a significant subset of those who experience public stigma, the process of self-stigmatization does indeed take place and this process is an element in the social construction of the addiction condition itself. 2 The burden of the paper is to explain and defend this claim, while recognizing its limits. These limits fit well with what Corrigan and Watson (2002: 36) have claimed in relation to what they call the “fundamental paradox of self-stigma” as this applies to those with a mental illness. They point out that there may be three types of response to public stigma. In addition to the group for whom public stigma leads to losses in self-esteem (the group of interest here), there are those who respond to stigmatizing prejudice with righteous indignation or even anger, and there are those who are simply indifferent to the treatment they receive as an out-group. We do not have figures on how these subgroups map onto the population of individuals affected by addiction, however, we take it as very plausible that these distinctions apply also in this domain, and so, given this, appropriate limits are placed on the scope of the proposed link between public and private stigma in the condition of addiction.^3^

Our central claim is supported by a study undertaken by the authors.^4^ The primary broader aim of the study was to investigate the impact of addiction on the moral self-conception, practical identity, and values of people in treatment for substance use problems. Material from the qualitative component of the study supports the view that affected individuals’ perceptions of public stigma feed into their (normative) self-conception. The case of most interest in the present context occurs when the person experiencing substance use problems identifies with the negative stereotype(s) of “addict” and related terms.

Our intention here is to make the case for how it is that there can be a link between public stigma and the development of the shame condition. Our thesis can be stated this way: *public stigma figures in the social construction of addiction in a significant range of cases*. The idea is that when public stigma is internalized by the person experiencing addiction (as self-stigmatization) it is a source of the shame condition Flanagan identifies. We take as our project here to unpack the move from public stigmatization to internalization of that stigma. Seeing how that process works will thus provide some support for, and understanding of, the social constructivist account.

To be clear, then, our main claim, that public stigma is an element in the constitution of addiction in a significant range of cases, is best situated within the literature that sees the phenomenon of addiction as socially constructed. Here, addiction is understood as the product of the interaction of substance, biology, individuals, settings of use, discourses, practices, and policies. It is historically and socially contingent, emerging through rather than preceding people’s and society’s understandings and experiences of it (Fraser, Moore, and Keane 2014; Granfield and Reinarman 2015). There are several studies that highlight the social situated-ness of addiction. A background starting point for these studies is the work done by Alexander and colleagues who noticed the connection between social conditions and conduciveness by rats to self-administer drugs—the famous Rat Park experiments (Alexander, Coambs, and Hadaway 1978). Then there are the studies done by Robins on Vietnam veterans who had used heroin extensively and regularly in Vietnam, yet ceased all substance use when back in the United States (Robins 1974, 1993); or the recent studies by Hart and colleagues on cocaine and opioid users in poor neighbourhoods where public stigma and police discrimination feeds into the lived experiences of addiction (Hart and Krauss 2008; Hart et al. 2000; Hart 2013). Of course we are not claiming that the internalization of stigma by some individuals is the only link between socially toxic circumstances and addiction experiences but rather that this process is central and important, and understanding how it plays out is important to any account of addiction within this tradition.

### Stereotyping and Self-stigmatization

It is a truism that social persons judge one another, interpret and evaluate each other’s behaviour, and find ways inevitably to group each other into ready-made normative categories. In stranger–stranger encounters we tag persons into types based on how they present, filtered through our own readily available stock of characters. This takes place perhaps pending the addition of further information that might fill out their actual social identity. But sometimes further facts about this person are not forthcoming, and we then proceed in our social interactions with an information-poor picture of the person before us. Of course the process of tagged group identification occurs spontaneously and heuristically as an understandable effect of facilitating social interaction. Sometimes, for instance, it quite inoffensively makes sense to read off the character or role of a person from their self-presentation, even if just as an ice-breaker in conversation. When, for instance, I wear my team insignia I do not feel in the least bit pigeonholed by the person who assumes that I am a sports fan of a certain type; and the same is true across a range of type-castings for getting an initial fix on who I am. Alas, this is not always the case, and so what might normally be a harmless and useful social process becomes corrupted when the categories become negative stereotypes and especially when those stereotypes are highly misleading representations of their members.

Stereotypes, as we will use the concept, are memetic categories that are supposed to characterize (“typify”) a group or individual and are based on simplistic generalizations. Their transmission through a culture occurs because the meme tends to go unchallenged and because of its fittingness with other cultural categories. Our use of “stereotype” dissociates from any possibly deserved moral attribution. So “negative stereotype” may involve an attribution of disapproval, but this leaves open any question concerning whether this disapproval is justified. Almost any group or individual can be the target of stereotyping, even apparently laudable groups such as those in the professions. The act of stereotyping relativizes to groups making these attributions, and usually the groups with powerful influence over public information are the most successful at promulgating their favoured memes. So, for instance in certain social quarters being a “greenie” is a negative stereotype, but the category of environmentalist necessary to it is arguably morally laudable. It is important to the current account that the stereotype of “addict” be understood in the way just described, viz., as a category based on a simplified generalization, tending to be spread by those with an interest in its preservation and yet, as we claim, giving rise to no implication of wrongdoing, moral badness, or weakness.

The negative stereotype associated with addiction comes about from public stigmatization of addicted persons. Comments by some of our respondents illustrate their awareness of its dangers:

I mean there’s a time in my life where I’d be paranoid about sitting around other people’s possessions you know ‘cause if anything went missing generally nine out of ten people in the room would be dismissed and I’d get the blame ... there’s a lot of discomfort within yourself after coming out of that lifestyle or existence really. —Tom^5^

I think the further that you go into addiction the further that, you know, you’re labelled and you’re stigmatized by being an addict. And the further that you go into addiction the harder it is to get out. —Bill

How easy is it for people to access this [the interview transcript]? Like does it go into a vault or whatever? Do you know what I mean? You know how people do their doc … they do essays and all that sort of thing and do they go into this? ... Yeah. People get so easily stigmatized, that would be horrible. —Brenda

The process of self-stigmatization is pronounced in addiction (Lloyd 2013; Luoma et al. 2007). It comes about via internalization of the negative stereotype, a resultant loss of self-esteem, and acting out of the negative public image. This public image excludes affected individuals from public engagement by seeing them as, for example, unreliable or untrustworthy. Affected individuals will then exclude themselves from public life, for example, by failing to apply for work or by removing themselves from public sight; or they will cease to see themselves as responsible citizens; or they will begin to see themselves as legitimate objects of the treatment meted out to them. Above all, they will be motivated to continue to consume *in order to forget, set aside, or reduce the negative feelings arising from their shame*. This is an instance of what, following Hacking (1995a, 1995b) we refer to as a *looping effect*. The normatively loaded classification of a group—in this case “addicts”—feeds back into behaviour that exhibits the classification. In this sense public stigma of addiction has the unfortunate tendency to feed into, sustain, or exacerbate the very practices it sets out to reproach.

## Flanagan’s Shame Condition

As noted above Owen Flanagan (2013) has recently proposed a shame condition as part of what he calls a twin normative failure model of addiction. In addition to the normative failure of effective agency and loss of control (common to most accounts), the affected person, in so far as she recognizes her repeated failures, “ … cannot pass her own survey” (this slightly unusual formulation simply means that the agent recognizes her own failures of effective agency, failures that thwart “the hopes, expectations, standards, and ideals she has for a good life ... ”)(Flanagan 2013, 1). In not passing her own survey, Flanagan says, she is bewildered and disappointed, and in particular the shame generated by recognition of her failures leads to “desperation and motivation to heal” (1). Flanagan intends his shame account “to describe normal and reliable features of addiction” (1). The exceptions he says include certain co-morbidity cases in which psychopathology accompanies addiction (such as those experiencing mania), people with access to their drug who have “no other choice-worthy options,” and the rich untroubled adventurous substance user who may revel in the lifestyle, a “Richard Burton, Richard Harris, Peter O’Toole … Christopher Hitchens [or] Keith Richards type” (8).

For convenience we will call Flanagan’s condition the shame condition, but really it is a condition with wider remit and includes other negative emotions and self-focused attitudes, for instance guilt. Now because our focus is on the link between public stigma and the shame condition, we will not spend time giving our own preferred elaboration of what is at stake in the shame condition; however, a comment is needed about the complex nature of guilt and shame in addiction. Shame is directed towards one’s whole self, whereas guilt is a feeling of culpability for an action. The shame of one’s addiction is then compounded by the aggregation of perceived guilt. Obviously this claim highly simplifies matters; for instance often the guilt or regret comes only after an affected individual reflects on what they have done. As Marc Lewis (2011, 191) puts it in relation to a period of his own addiction:

… I could not escape the shadows of guilt and shame for very long. The voices that I imagined scolding me, haranguing me, came from inside after all. They fed off my own angry shock at the lengths I was apparently willing to travel in order to feel potent and strong, free and resplendent. I continued to rebuild my own prison, taking chances that showed a terrible disregard for my safety, my life, leading back to a small, dark pool of anger and despair …

And as one of our own study respondents put it:

[A]t the end of the day, you’ve got to be happy with yourself and I’m not happy with myself, I don’t look in the mirror and say I love myself, I don’t even like myself, I self-loathe myself, I hate myself, I hate what I’ve done to myself and done to others by doing it to myself. —Tim

By including shame among the conditions of addiction the theorist does not thereby subscribe to the so-called moral model of addiction. (Flanagan [2013] denies that his own account commits him to “moralizing addiction”). The reason for this is twofold: first, the *emotion* of shame is often cut loose from the conditions that would warrant genuine moral censure. Second, shame is broader than a sense of culpability or “guilty mind” condition in the criminal law (*mens rea*). One may experience shame in many types of blame-free conditions. All that is required (in the typical cases) is a failure to present or compose oneself in a manner deemed appropriate by oneself or others or a failure to publicly display one’s agency uncompromised by external or incidental interferences, or to present oneself as socially different on account of some physical feature, deformity, cultural, or religious presentation.

Take for instance bodily self-presentation. For the affected individual on the street this can be significantly disabling. There is no culture we know in which the mainstream celebrates deviant bodies, and indeed western cultures typically emphasise bodies that are strong, young, and healthy. Having an illness often makes people feel ashamed, although they have done nothing to be ashamed of. We imagine how we compare with others, with our former selves, or with the prevailing normative standards. With the loss of control over our body, we lose control over an image that defines us, and we lose control over what is tacitly, sometimes even explicitly, a carefully chosen self-presentation. At these times we try to diminish this unwanted visibility, we stay in during bad days, we cancel visits from friends, we try to control the information our body sends out.

The experience of shame dissociates from an affected individual’s sense of blameworthiness so that shameful actions need not be immoral actions. This bears further elaboration. The shame condition obtains independently from either a sense the affected person has of doing the wrong thing or of what is wrong. Of course, many affected individuals are ashamed of their addiction and what they do in that capacity, and so the shame of addiction is causally related to their sense that addiction is socially discreditable. They frequently blame themselves for it as well. On the other hand, many affected persons do not feel as though they are to blame, either for being addicted or for many of the things they have done. Nevertheless they will be motivated to disguise their addiction in public because it compromises them as social persons.^6^

In a closely related point David Velleman (2001, 44) explains that one can feel shame “without being ashamed of anything in particular.” He gives the example of the shame teenagers experience on account of being seen by their peers in public accompanying their parents. They do not think their own parents are “especially discreditable as parents” (2001, 44), and so the source of the shame is not *them*. So, true, there is no specific object grounding the shame experience, but nevertheless, and as Velleman sets out, there is a perfectly good explanation for the experience having to do with the teenage efforts at giving birth to an adult social identity, and those efforts entail the need to erase the presence of the childlike social identity. This seems exactly right and it has its analogue in the story we are telling about addiction. To the extent that one is compromised by presenting socially with an addiction identity it is disabling to one’s social agency. One need not believe one’s compulsive consumption is discreditable in order to experience the shame that comes from addiction, but the knowledge that others will discredit those they have marked out as (for example) low types, is often sufficient for one to erase or hide one’s addiction identity in public.

## The Link Between the Shame Condition and Public Stigma

We think the sources of the shame condition typically go beyond the affected person’s inability to be an effective reasons-responsive agent, someone who passes *her own* survey, which makes the source of addiction’s shame look too narrow. We think it crucial to locate a central source of addictive shame in connection to the truism of stereotyping mentioned earlier: the fact that social persons judge one another, interpret and evaluate each other’s behaviour, and find ways inevitably to group each other into ready-made normative categories, in this case the negative stereotypical category of “addict,” “junkie,” “ freak,” “fiend,” “user,” “druggie,” “dopehead,” and so on. Not every use of these terms conjures negative feelings to be sure—especially when a term’s pejorative sting is neutralized via in-group adoption—but it is the connotation of exclusion-for-being-a-bad-person that carries negative weight. And when the affected person assents to the stigmatized content (either through reflection or implicitly), they have at that point internalized it, and this leads to a kind of self-accusation. For not only have they failed self-examination, they regard themselves as being the subject of *society’s* survey, and here they fail as well. The internalization of public stigma gives rise not just to losses in esteem, it is revealed also in attempts to conceal an addiction identity, for example, in reticence to pursue work or to undertake independent living opportunities (Corrigan and Watson 2002, 38).

We do need to be careful in how we characterize the translation between public stigma and internalization of the negative beliefs to the stigmatized addict. Corrigan and Watson put the point in terms of characterizing self-stigma using the concepts of public stigma that are mirrored within the individual (2002, 38). We think at certain points such mirroring of concepts must be understood only instrumentally. The origin of the concept of stigma has it as an interpersonal process or more accurately a process in which those with power and authority in a social system direct others to be marked out for identification and careful treatment. In the case of a person who is stigmatized by others, and who then, accepting this treatment, stigmatizes herself, the self-prejudice and self-discrimination that follows is real enough but only once we correct for differences in process between the inter-personal case and the intra-personal case. Obviously I cannot refuse to give myself the job I have advertised, refuse to give money to myself as I beg in the street, or refuse myself the accommodation I have just advertised and applied for. Nevertheless, I can self-sabotage in multiple ways that make my life go a lot worse precisely because I have come to agree with society’s mark on me which says “I am not a worthy person to participate in social life.” It is an important and non-trivial exercise to analyse the moral psychology of the self-stigmatized to investigate the multiple ways in which such self-sabotage plays out. Comments by some of our respondents illustrate the generative role of self-stigma in producing self-sabotage:

I struggle [with] people offering me help, I still think that I’m not worthy of it ’cause they ... everyone’s been offering me to help move and I said “no, no, it’s alright man I’ll get a taxi or I’ll carry it or whatever,” and yeah the guy … the guy at [treatment service] said the other day he sees it as me being … me myself thinking I’m not worthy of anyone’s help. —Graham

Once we start something good we feel guilty because we feel like we don’t deserve it. —Diane

Other respondents explicitly described their behaviour as self-sabotage:

I know a lot about all of these drugs, I know how to get off them, I know treatments, I know all that, I know as much as the doctors sometimes, don’t get me wrong and people think … sometimes people think addicts are stupid dumb idiots and that, a lot of us … I’ve met a lot of really intelligent addicts, you know, so I don’t think that at all, I know I’ve got potential as well but there’s just something in me that, yeah, keeps self-sabotaging. —Tim

I think it’s self-sabotage, every time I nearly get somewhere I fuck it up and I don’t deliberately do it but that’s what happens and I … sort of realized every time I almost get somewhere something dramatic happens or I’m a drama queen in some way and I don’t know what it is, it’s fear I think, fear of whether or not I can hold it together to do something because if I fail at one more thing I’ll just be even more ashamed of what I can’t do. —Nicole

It is remarkable that in their descriptions of self-sabotaging behaviour, these respondents also include negative judgements about their identity (“people think addicts are stupid dumb idiots,” “I’m a drama queen”), highlighting the close link between self-sabotage, negative self-image, and self-stigma. These, in turn, further consolidate the negativity attaching to the addict stereotype and concomitant disruption to normative agency.

## A Compounding Effect of Stigma in Addiction

In this section we explain the link between public stigma and shame in terms of a compounding effect of two features of addiction the public respond negatively towards. To do this we may compare the claims about shame made above to Erving Goffman’s three contexts for stigma (1963, 4). He cites, first, “various physical deformities,” second, “blemishes of individual character” (he mentions for example mental disorder, imprisonment, addiction, unemployment, radicalism), and third the “tribal stigma of race, nation, and religion.” It is clear that addiction self-stigmatization is located within the territory of the second grouping (indeed, Goffman explicitly says so), but we would add that many self-presenting substance users are *physically* marked.^7^ They, as it were, bear the bodily signs exposing them to (often) moralistically motivated evaluation as weak, and bad, and so compromised in their social standing. This doubling up of markings in some cases is an exacerbation of the conditions leading to self-stigmatization, and that is because a physical marking is an advertisement for the second grouping. It is a sign to the social world that the substance user is weak or bad both on their face and throughout, reaching inwards to a corrupted character.^8^ It removes the possibility of concealment, and thereby reduces the availability of a key technique that protects privacy, a zone where these signs might otherwise be under the control of the person. Thus physical marking (a stigma in itself) points at or advertises character blemish (a second stigma), thereby compounding the overall effect on the shamed agent. The following comments by respondents, describing physical markings and blemishes of character attaching to their substance use, are illustrative.

I look at my arms and I think God blimey, who wants to go out with that? … Heroin doesn’t leave those kind of marks, that’s ice. (…) But that affects me, do you know what I mean? Like I can’t wear short tops, I can’t … just can’t be a normal person anymore. —Isobel

I just want to be able to do what everyone else does, (…) and unfortunately I’ve got marks from my using (…) if I was doing customer service for example a doctor would know that I used to use and I don’t know if it would help me get a job, I’d have to wear long sleeves every day and there’s a lot of things I’d have to do to make myself feel presentable enough. —Martina

… [i]n my area, like you’re a marked person if they know you’re on methadone. —John

Sometimes I go outside and I really feel hated, … I thought it must be how I dress or [the] expression on my face or something. You just constantly feel like you’ve got a big neon sign on your head saying “loser,” you know, “contemptible loser.” So when someone actually … in a shop or something they’ll actually smile at you or act like you’re a normal being, human being, it’s really restorative, it cheers me up for days. —Lachlan

## Shame and Guilt as Counterproductive Emotions

Flanagan claims that feelings of shame motivate the healing process (2013). This might seem to imply that self-stigmatization leads to treatment-seeking and even recovery (although we are not saying that Flanagan makes this bolder claim). There is *some* evidence for this but such a claim is too broad and too swift. The elimination of public stigma, we claim, would, in the first instance, greatly alleviate the experience of addiction in many cases, but we do not think that eliminating public stigma would also eliminate the motivation an affected person has to address their vulnerable status as someone locked into a seemingly interminable pattern of consumption. To put this in Flanagan’s terms, the removal of public stigma would dissolve the pressure to pass society’s survey, but the pressure to pass one’s own survey, to live up to one’s own standards, might well remain.

Often when we realize that what we are doing goes against what we regard as the correct course of action, one that fits with ideas of who we really are or should be, we do then sometimes feel shame and are motivated to live in accordance with our values again. Some studies do support the claim that stigmatization of people will motivate them to seek treatment and in that narrow sense stigmatizing people will lead to recovery (Bayer 2008). But other studies have led to doubts on this score. The question is whether the positive effects of treatment motivated by shame—which often fail to endure—outweigh the negative effects of public stigma. For public stigmatization is long-lasting, pervasive, and often inescapable; over time, it undermines confidence, trust, and the capacity to form supportive relationships (Williamson et al. 2014). Stigma has been associated with diminished self-esteem and self-efficacy and significantly interferes with a person’s life goals and quality of life (Corrigan and Watson 2002, 35, 39). In this sense, feelings of shame are counterproductive, leading to a quality of life that undermines the motivation needed to heal.

That individuals who identify as addicted have negative self-regarding feelings leading to drug use is well-supported by our qualitative data. Mostly feelings of remorse motivate change for the better, but after failing to improve one’s life time after time, aggregated feelings of guilt come to reinforce use. These feelings are then seen as salient factors in the push to use. Moreover, such feelings begin to “bond” with the motivation to consume so that the effect of using, ironically, is to blunt or eradicate the negative self-regarding feelings and attitudes of one’s use. In these examples, the shame of use turns out to be cyclical and self-perpetuating. In our study, we came across a preponderance of examples in which some version of this looping effect was displayed. When asked how he reacted when he did something he regretted, one respondent replied: “Probably got drunk.” Other respondents drew similarly explicit links between negative self-regarding feelings and substance use:

I know a lot of my heavy using was because I was ashamed of what I was doing and it didn’t … commonsense approach would be to not use. But in my case, it was, use more so I could forget how bad I was feeling about myself. —Brigitte

I’d stuffed up so many times with things. That’s why I drunk as well, it wasn’t to self harm myself, it was just to, like I say, get drunk and stop thinking about what I’d done wrong and where I went wrong. —Frank

I wake up in the morning and go oh what have I done, oh I’ll just have another drink. —Simon

Yeah oh it’s just constantly in the back of your head and that’s just even more of an excuse to drink and to just eliminate that or just for it to go away for a while but then the next morning or when you wake up sober and it’s there ten times as worse and it’s just like a revolving circle. —Peter

## Shame and Diagnosis

Our thesis fits neatly with the two standard international nosological tools for substance-related disorders, viz., the American Psychiatric Association’s (APA) *Diagnostic and Statistical Manual* (DSM) (2000/2013) and the World Health Organization’s (WHO) *International Classification of Diseases* (ICD) (1992). The DSM and ICD systems achieve the diagnosis of addiction by first establishing a general definition and then by providing more detailed subsets of symptoms in which severity of disease depends on the satisfaction of some threshold. In the DSM-5 (American Psychiatric Association 2013) the presence of just two to three symptoms would lead to a diagnosis of a mild addiction. The social impairment grouping (symptoms 5–7) and the impaired control grouping (1–4) in particular make explicit reference to normative standards. Consider:

- 3. A great deal of time is spent in activities necessary to obtain …, use …, or recover from its effects
- 5. Recurrent … use resulting in a failure to fulfil major role obligations at work, school, or home
- 6. Continued … use despite having persistent or recurrent social or interpersonal problems caused or exacerbated by the effects of [the substance]
- 7. Important social, occupational, or recreational activities are given up or reduced because of … use

Excluding the physiological criteria of tolerance and withdrawal, then, these defining symptoms of addiction already rely on normative assumptions about appropriate standards of behaviour (Fraser, Moore, and Keane 2014). These standards include self-control (5, 7), rationality (6, 7), responsibility (5, 7), and appropriate use of time (3, 7). To be diagnosed with addiction under these systems then, is to be classified as morally compromised or deficient. To take on the classification, to self-identify as an addict, is to take up the associated self-stigma of this moral subject position. Additionally, the inclusion of the symptom of craving or compulsion to use (American Psychiatric Association 2013; World Health Organization 1992) means that the person diagnosed with addiction typically sees himself or herself as giving in to a temptation to use and so (justifiably or not) as responsible for the failures and problems specified in the social grouping.

Moreover, it is not only scientific diagnostic systems that build in a normative condition leading to the possibility of self-stigma. The constitutive role of self-stigmatization in addiction is also apparent in the twelve steps of Alcoholics Anonymous. Here, seven of the twelve steps require the person to identify and acknowledge their failures: admitting “wrongs,” “defects of character,” and “shortcomings” and making amends for harms to others (Bill W. 2001). In the twelve-step disease model of addiction, these normative failures are assumed as characteristic of all addicted persons and steps towards “recovery” demand acceptance of these as part of one’s addict identity. (It is noteworthy that Flanagan’s condition is compatible with his apparent acceptance of the twelve-step model.)

Ian Hacking’s (1995a) ideas on the looping effects of human kinds provide a useful approach to thinking about the ways in which the normalizing processes inherent in addiction diagnosis produce self-stigma. Addicted persons may be seen as a human kind—a classification of people constituted by “generalizations sufficiently strong that they seem like laws about people, their actions, or their sentiments” (1995b, 352). Addicted human kinds are constituted through particular historically and socially situated concepts of the disease of addiction and its defining symptoms. These concepts, as we have shown, are loaded with moral values. Hence, the *classification* as an addicted human kind is loaded with moral value. The looping effects of Hacking’s thesis refer to the processes whereby “people classified in a certain way tend to grow into the ways they are described.” Taking up a morally loaded addiction identity, then, would, for many, demand taking up the self-stigmatization we have identified.

## Recovery Systems

Some further suggestive evidence that self-stigmatization plays the role we have identified is provided by the experiences of many in treatment. Some of our respondents described the turning point in their addiction in terms of self-acceptance; in other words, this turning point was accompanied by a cessation in the process of self-stigmatization (we say “accompanied by,” because we are pointing to some empirical evidence for our thesis, not a failsafe proof of it). In this connection it is worth detailing one of our cases to see how it may play out.

Alice used marijuana and LSD as a teenager, dropped out of school and ran away from home. She formed a partnership with a heroin dealer and became dependent on heroin and for many years lived a turbulent life both on and off the streets, in and out of prostitution. She later had two daughters and became fearful that the stigma of drug addiction would affect them:

[F]or years when my kids were young I was always worried about other people finding out that I was an addict and … I was worried about it being taken out on the kids. Oh your mum’s a junkie you know.

After the birth of her second daughter, Alice started on methadone and for a brief period—a couple of years—she maintained a home with her children and a new partner. Following a series of personal tragedies, however, Alice had a mental breakdown and her children were placed into foster care. Separated from her children for the next ten years, she became homeless again, recommenced heroin use, began to drink heavily, and also became addicted to valium and methamphetamine (“ice”). The turning point in her life was when she was in her late thirties and was reunited with her children and given a second chance by her father and step-mother. Her daughters told her “mum we love you the way you are.” Her parents said to her: “if you need to have a drink every day ... have your methadone every day to lead a normal life … then so be it.” It is highly plausible that this acceptance from her children and parents provided an important basis for the removal of Alice’s self-stigmatization. The acceptance of herself is captured in her own words:

I’ve come to a point in my life where I can’t say I’m proud of what I’ve done or anything, but I’ve accepted it and I’m okay with who I am. It’s taken me … like I’m 45 now. It’s taken a long time, … and a lot of that had to do with the stigma of being homeless and being a drug addict and all of that … I wouldn’t go so far as proud, but I’m happy with myself … it’s taken me ’til 45, but … I’m finally starting to do things that are productive, that aren’t counterproductive.

A plausible interpretation of Alice’s re-evaluation of her situation is that insofar as she was released from prior feelings of guilt and shame she was greatly helped in maintaining control over her substance use. (This reading of Alice’s case—that the removal of stigma had a decisive stabilizing effect on her self-control—was how one of the author’s of the study in particular interpreted the outcome. In Alice’s case, interviews were done over successive years of the entire study, providing a robust degree of continuity.)

The case of Alice is representative of a type of response exhibited by other respondents, for example:

I don’t need to use anymore, ‘cause I like myself, who I am. —Sarah

And this one:

R: (Pause) The best thing, I guess, is that I’m still alive. Yeah, and I’m … I seem to be … I’m not as hopeless as I was last year. (…)

I: And what changed, do you think?

R: Mm. Acceptance … ( … ) that my marriage was over and that there was a distinct possibility I may be on methadone or a drug replacement for the rest of my life. There is that possibility. Yeah, I think just acceptance. Accepting who I am. —Nick

An important qualification to the claim that self-acceptance blunts self-stigma involves the recognition that affected persons (in a non-use phase) can occasionally consume substances without also engaging in self-reproach. Self-acceptance includes some self-knowledge of vulnerability and a self-directed forgiving attitude. By adopting this attitude further resilience against the tendency towards shame may obtain. The desire to consume substances can then be seen as a non-self-stigmatizing part of one’s identity. The following respondents exhibited this attitude quite strongly:

Yeah I think acceptance has got a lot to do with that for me … I had to start a new life ’cause I tried changing my life so many times by stopping … and my new life is an abstinence-based life. I think that’s … for me, that’s acceptance. And not making grand statements like I’ll never use again because I mean that … yeah in my heart I think it’s my intention, but I’ve only got today. —Dan

… other times I sort of picked up the pieces and then I failed at a few things and I just went no, stuff it, I lost my place to live again and I was back to that … back to where I started, so that’s … the last time I went to rehab I said I make sure even if I do have a beer I’m not going to punish myself for it. —Paul

## Objection and Response

We have claimed that shame, or self-stigmatization, figures as a part-constituent of the dynamic process in which addition is formed. But this is a normative condition, and some might object that, as such, it cannot play the theoretical role we have assigned it. Isn’t addiction supposed to be some physical state of a human being? We reply by noting that to think addiction is merely a physical state of a person is to miss something important: addiction is an externalizing disorder, and so rather than a static condition, it must be understood dynamically in terms of how behaviour over time depends on responses to social conditions. It is not merely that addicted persons are physiologically changed but rather that these changes manifest in thinking and behaviour that is maladaptive for the social environment. Public stigma giving rise to shame amounts to recognition that one is not living up to the standards of one’s social group, standards that one buys into, and so one will be disposed in manifold ways to correct this. By getting well, and by that very process, a person will detach themselves from the source of public stigma.

To bring this out, contrast the typical western case of a regular opiate user with the very interesting case of a regular opiate user in the north-western Indian state of Rajasthan. During the Riyan (opium) ceremony guests are invited to consume a small amount of opium in return for friendship. This is an old tradition in which bonding between parties is enabled all within a ritualistic frame, with specific social rules determining the process. For example, refusal to consume is viewed as an insult. Thus we have consumption of opium taking place within a normative framework that culturally endorses the activity. Given the absence of stigma and shame, could there be addiction here?

The answer is complex. On the one hand we can imagine that regular participants might experience mild withdrawal, but in the case where regular consumption takes place with a steady supply of opium, no withdrawal is experienced, and life may go on. The negative consequences of the biological effects of the opium are not present. Neither of the twin normative failures Flanagan mentions is present. Specifically, no public stigma obtains, and so no self-directed shame obtains either. Still, the biological elements of repeated consumption may be present in the form of physical withdrawal symptoms and neuro-biological adaptations. So what are we to say in relation to the question of addiction? Perhaps we should begin by noting that we appear to be in possession of all of the relevant social and biological facts pertaining to the example. The brain adaptations are present and manifest in behaviour that is contextually driven by the socio-cultural norms. Perhaps related to the Riyan ceremony are cases in which consumption outstrips the ritual requirements; but let’s bracket such cases, and let’s imagine for a moment that no negative social consequences arise. Then plausibly, no public stigma is present, and the shame condition is nowhere to be seen. On a social constructivist account there is no addiction here then. For the apparent drive to use opium in this context is not felt as a motivation with a negative valence. Moreover the desire to consume is accompanied by a social condition that carries the weight of implicit endorsement. What we have here is a case of collective willing consumption. So partakers can be in possession of a very strong desire to take opium while recognizing (again perhaps implicitly) that this is a desire which is socially endorsed.

A final comment here is in order concerning the alleged stigma-removing effects advocated by the (neurobiological) disease accounts of addiction. The line of argument is often put that in so far as addiction is seen as a chronic relapsing brain disease it will be seen as a condition that the addicted person cannot really control and so a condition that ought not attract blame, censure, reprimand, and so on. There is *some* evidence that this is the case, but as Buchman and Reiner (2009), 18–19) point out there are some unintended consequences to which this move gives rise. One effect, they say, is encouragement to acceptance of an “us–them” distinction (the normal and the diseased); another is that the label of diseased addict creates perceptions of dangerousness and unpredictability; and another is the self-labelling effects of having “a different *kind* of brain.” Social construction theory again is helpful in seeing that in attempting to translate neuroscience research for the public, there are interpretative filters that, as they put it, “ … may inadvertently contribute to the beliefs that perpetuate stigmatizing attitudes” (19).

## Conclusion

The thesis of this paper is that public stigmatization of addiction has a private correlate: internalization of the social opprobrium attaching to the negative stereotype of addiction leads to looping effects on behaviour. Evidence for our claim, taken from a qualitative study with addicted persons themselves, points to some direct effects of this public stigmatization, including those cases where a pattern of consumption comes to be directly motivated by the need to forget, erase, or avoid the shame of addiction itself. We make no claims regarding the extent of the looping effects we have identified. An empirical test for that would be to observe social environments that mimic social environments in which addictions are present but where stigmatizing attitudes are largely absent. In this situation, if we are correct, one would predict a diminution in the severity of addiction, or an absence altogether, commensurate with an absence of stigma in the relevant cases—the ones in which agents internalize the stereotype and so give effect to the looping mechanisms we have outlined here. If this is right, there are implications for social policy and for programmes designed to alter attitudes towards individuals experiencing addiction, their behaviour, and the way they present in social life (Patterson and Keefe 2008, 122).

## Footnotes

1 For our purposes here the shame condition and the self-stigmatization condition are the same, and we will use these terms interchangeably. As we have just outlined the sources for them can be narrow (say in the case where a person experiencing addiction fails despite being given public support), or more generally these sources can be social and public (say in the case where one internalizes the negative stereotype). We focus in this paper exclusively on the latter case where shame (self-stigmatization) derives from public stigmatization.

2 Social construction theory is an ontological theory that contends that reality is socially constructed. In this view, phenomena—including addiction—do not exist objectively but are the product of discourses and social and cultural forces. Social constructivism is best understood as a dynamic theory in which ideas and concepts are externalized, then objectivized, and finally internalized by social actors (Berger and Luckmann 1966). In the present case, the phenomenon of addiction—the ideas, activities, and objects associated with addiction and the state of addiction itself—is objectified and reified, turned into a “thing” and externalized as existing outside the unique and varying experiences of particular individuals.

3 As we go on to outline, our thesis is supported by the qualitative component of a study on addiction and moral identity and agency. We are unable to determine from this material the extent to which Corrigan and Watson’s self-esteem category is represented in the overall population of people affected by addiction.

4 The study, funded by the Australian Research Council (DP1094144), employed a mixed methods longitudinal design. We followed up three main groups (people in treatment for problems with alcohol, people in treatment for problems with opioids, and a comparison group: alcohol and other drug workers) over a four-year period (baseline in 2012 and successive 12 month follow-up episodes in 2013, 2014, and 2015). All study participants (n=242) completed a structured baseline questionnaire. A subset of study participants also completed an in-depth interview about how they saw their life, their capacities for self-control, and their substance use. Participants in this subset were followed up and re-interviewed over the four-year period. In this paper we draw on data from the qualitative (in-depth) interviews conducted with people in mainly heroin or alcohol treatment (n=69from the Sydney node of the study, n=40 from the Melbourne node). Tiny proportions from the qualitative component were methamphetamine or poly-drug users.

5 Pseudonyms are used here and elsewhere for all study participant quotes, although gender has been preserved.

6 Interestingly the phenomenon of undeserved public shame has been described in the bioethics literature on disability. See for example the work of Havi Carel (2008), Charmaz and Rosenfeld (2006), and Toombs (2001).

7 Two important clarifications should be made here. First, again, care needs to be taken in linking self-presentation and stigma, for we also risk buying into the stereotype of addict that views this type as a poor, homeless, physically damaged individual. The financially well off professionally employed person who injects drugs enjoys relative anonymity which shields her from the harm of public stigma. Still, the distinction is in some ways grist to our mill: this person disguises their substance use precisely to avoid the stigma that might well lead to catastrophic exclusion from privileged life and the negative consequences of an addict identity. Second, although for space reasons we do not discuss it, the stigma of race—one of Goffman’s tribal stigmas—also can play as an exacerbating feature in this process. See, for example, Carl Hart (2013) who elaborates the institutional and racial stigmatization of addiction.

8 We emphasize of course that we are offering an analysis of how stigma works in the case of addiction. As noted earlier we have no truck with the so-called moral model of addiction. Public stigma of persons affected by addiction is generated by a host of unjust and uninformed moralizing that is damaging to people experiencing difficult life circumstances.

## References

[CR1] Alexander BK, Coambs RB, Hadaway PF (1978). The effect of housing and gender on morphine self-administration in rats. Psychopharmacology.

[CR2] American Psychiatric Association [APA] (2000). *Diagnostic and statistical manual of mental disorders DSM-IV-TR*.

[CR3] American Psychiatric Association [APA] (2013). *Diagnostic and statistical manual of mental disorders DSM-5*.

[CR4] Bayer R (2008). Stigma and the ethics of public health: Not can we but should we. Social Science and Medicine.

[CR5] Berger PL, Luckmann T (1966). *The social construction of reality*.

[CR6] Bill W (2001). Chapter 5: How it works. *Alcoholics Anonymous (PDF),* 4th ed.

[CR7] Buchman, D., and P.B. Reiner. 2009. Stigma and addiction: Being and becoming. *The American Journal of Bioethics—Neuroscience* 9(9): 18–19. 10.1080/15265160903090066 PMC2791913 19998183

[CR8] Carel H (2008). *Illness. The cry of the flesh*.

[CR9] Charmaz K, Rosenfeld D, Waskul DD, Vannini P (2006). Reflections on the body, images of self: Visibility and invisibility in chronic illness and disability. *Body/embodiment: Symbolic Interaction and the Sociology of the Body*.

[CR10] Corrigan PW, Watson AC (2002). The paradox of self-stigma and mental illness. Clinical Psychology: Science and Practice.

[CR11] Flanagan O (2013). The shame of addiction. Frontiers in Psychiatry.

[CR12] Fraser S, Moore D, Keane H (2014). *Habits: Remaking addiction*.

[CR13] Goffman E (1963). *Stigma; notes on the management of spoiled identity*. *A Spectrum book*.

[CR14] Granfield R, Reinarman C (2015). *Expanding addiction: Critical essays*. New York.

[CR15] Hacking I (1995). *Rewriting the soul: Multiple personality and the sciences of memory*.

[CR16] ____. 1995b. The looping effects of human kinds. In *Causal cognition: A multidisciplinary debate*, edited by D. Sperber, D. Premack, and A.J. Premack, 351–394 New York: Oxford University Press.

[CR17] Hart CL, Haney M, Foltin RW, Fischman MW (2000). Alternative reinforcers differentially modify cocaine self-administration by humans. Behavioural Pharmacology.

[CR18] Hart C, Krauss RM (2008). Human drug addiction is more than faulty decision-making. Behavioral and Brain Sciences.

[CR19] Hart C (2013). *High price: A neuroscientist’s journey of self-discovery that challenges everything you know about drugs and society*.

[CR20] Lewis, M. 2011. *Memoirs of an addicted brain: A neuroscientist examines his former life on drugs*. New York: Public Affairs.

[CR21] Lloyd C (2013). The stigmatization of problem drug users: A narrative literature review. Drugs: Education, prevention and policy.

[CR22] Luoma JB, Twohig MP, Waltz T (2007). An investigation of stigma in individuals receiving treatment for substance abuse. Addictive Behaviors.

[CR23] Patterson DL, Keefe RH (2008). Using social construction theory as a foundation for macro-level interventions in communities impacted by HIV and addictions. Journal of Sociology and Social Welfare.

[CR24] Robins, L.N. 1974. *The Vietnam drug user returns*. Washington D.C.: U.S. Government Print.

[CR25] Robins LN (1993). Vietnam veterans ’ rapid recovery from heroin addiction : A fluke or normal expectation?. Addiction.

[CR26] Toombs KS, Toombs KS (2001). Reflections on bodily change: The lived experience of disability. *Handbook of phenomenology and medicine*.

[CR27] Velleman JD (2001). The genesis of shame. Philosophy and Public Affairs.

[CR28] Williamson L, Thom B, Stimson GV, Uhl A (2014). Stigma as a public health tool: Implications for health promotion and citizen involvement. The International Journal on Drug Policy.

[CR29] World Health Organization [WHO] (1992). *The ICD-10 classification of diseases and related health problems*.

